# Measurement of acetabular inclination and anteversion via CT generated 3D pelvic model

**DOI:** 10.1186/s12891-017-1714-y

**Published:** 2017-08-29

**Authors:** R. Y. Wang, W. H. Xu, X. C. Kong, L. Yang, S. H. Yang

**Affiliations:** 1 Department of Orthopaedics, Wuhan Union Hospital, Tongji Medical College, Huazhong University of Science and Technology, Wuhan, Hubei 430022 China; 2 Department of Radiology, Wuhan Union Hospital, Tongji Medical College, Huazhong University of Science and Technology, Wuhan, Hubei 430022 China

**Keywords:** Acetabula, Inclination, Anteversion, Computed tomography, Three-dimensional reconstruction

## Abstract

**Background:**

Inclination and anteversion were the main factors that determined the reliability of the acetabulum. Inclination and anteversion measurements included anatomical, operational and radiographic methods. The aim of our present study was to exhibit divergence of inclination and anteversion via the three measurements.

**Methods:**

Inclination and anteversion were defined according to the definitions put forward by Murray. Three-dimensional models of pelvis of CT data were brought forth. Acetabular axis was determined by the rim of acetabula. Reference planes were established by bone landmarks including anterior superior iliac spine, pubic tubercles and sacral crests. Inclinations and anteversions were calculated according to the definitions.

**Results:**

Forty-nine cases were involved in the research. Data of inclination form anatomical, operational and radiographic showed 37.48 ± 11.07, 45.12 ± 14.76 and 48.76 ± 14.36, and anteversion were 18.12 ± 7.59, 24.97 ± 9.68, 14.30 ± 5.64. A substantial deviation was noted in the inclinations (*P* < 0.01) and anteversions (*P* < 0.01).

**Conclusion:**

Our findings suggested that the inclinations and anteversions of the three measurements varied, which might in turn interfere the decision of orthopedists.

## Background

Total hip arthroplasty (THA) was considered the routine choice of treatment for osteonecrosis of femur head, osteoarthritis, developmental dysplasia of the hip (DDH), etc. Dislocation of the hip and aseptic loosening of prosthesis were the most frequent complications that soon occur after a THA [1]. The dislocation was one of the most critical factors that affected the quality of life of patients postoperatively [2]. Previous studies had demonstrated that a number of factors were involved in the mechanism of dislocation, which included head size of the prosthesis, cup size, cup-to-head ratio, leg-length discrepancy, cup inclination and anteversion, etc. [3, 4].

Inclination and anteversion were the most pivotal parameters that determined the quality of THA [5]. Inappropriate inclination and anteversion might cause dependent dislocation, and in turn cause femoroacetabular impingement syndrome (FAI) [6]. A higher inclination might cause hypo-cover of the hip component, which was defined as iatrogenic DDH, while a smaller inclination might cause FAI during abduction. A recognized range of inclination was from 40 to 45 degrees [7]. A larger anteversion might cause FAI during external rotation while a smaller anteversion might cause FAI during flexion and internal rotation of the hip. A recognized range of anteversion was from 15 to 20 degrees [8].

In 1993 Murray determined three methods of acetabular measurement, which includes anatomical, operational and radiographic measurements of inclination and anteversion [9]. Three measurements were applied in different situations. Operational measurement was commonly used in THA operations in lateral approach. Patients were placed in lateral position with the reference plane on the operation table in sagittal plane. Radiographic measurement was normally used in the anterior posterior X-Ray film [10]. Anatomical measurements were primarily used for corpses. As per the performance of direct anterior approach, they were the most important acetabular parameters considered during the operation [11]. The definitions of three measurements were outlined below. Radiographic measurement was applied in most researches based on the anterior-posterior film of the hip, which was two-dimensional in orientation [12], while anatomic measurement was applied in some researches using three-dimensional regeneration, which was three-dimensional in orientation [13, 14].

A few research has verified the three measurements put forward by Murray. Higgins confirmed the difference between anatomic, operational and radiographic measurements among American population, but the difference among population of central of China has not been investigated [15]. It is still unknown as to whether these differences could influence the operators in making decisions. Our research intended to investigate the differences among the three measurements within the same acetabula.

## Methods

### CT data collection

The research was approved by Ethics Committee of Tongji medical college, Huazhong University of Science and Technology. Patients according to the inclusion criteria were retrospectively included into the research. CT data of healthy cases were collected from imaging database of Wuhan Union Hospital from January to October in the year 2014. The inclusion criteria were bilateral iliac crest to proximal femur whose radiological diagnosis reported no disease. Patients with particular diseases such as pelvic fracture, ONFH, THA, DDH, ankylosing spondylitis as well as lumbar intervertebral fusion were excluded. Data that fit the criteria was saved in DICOM format.

### Definition of inclinations and anteversions

**Fig. 1:**
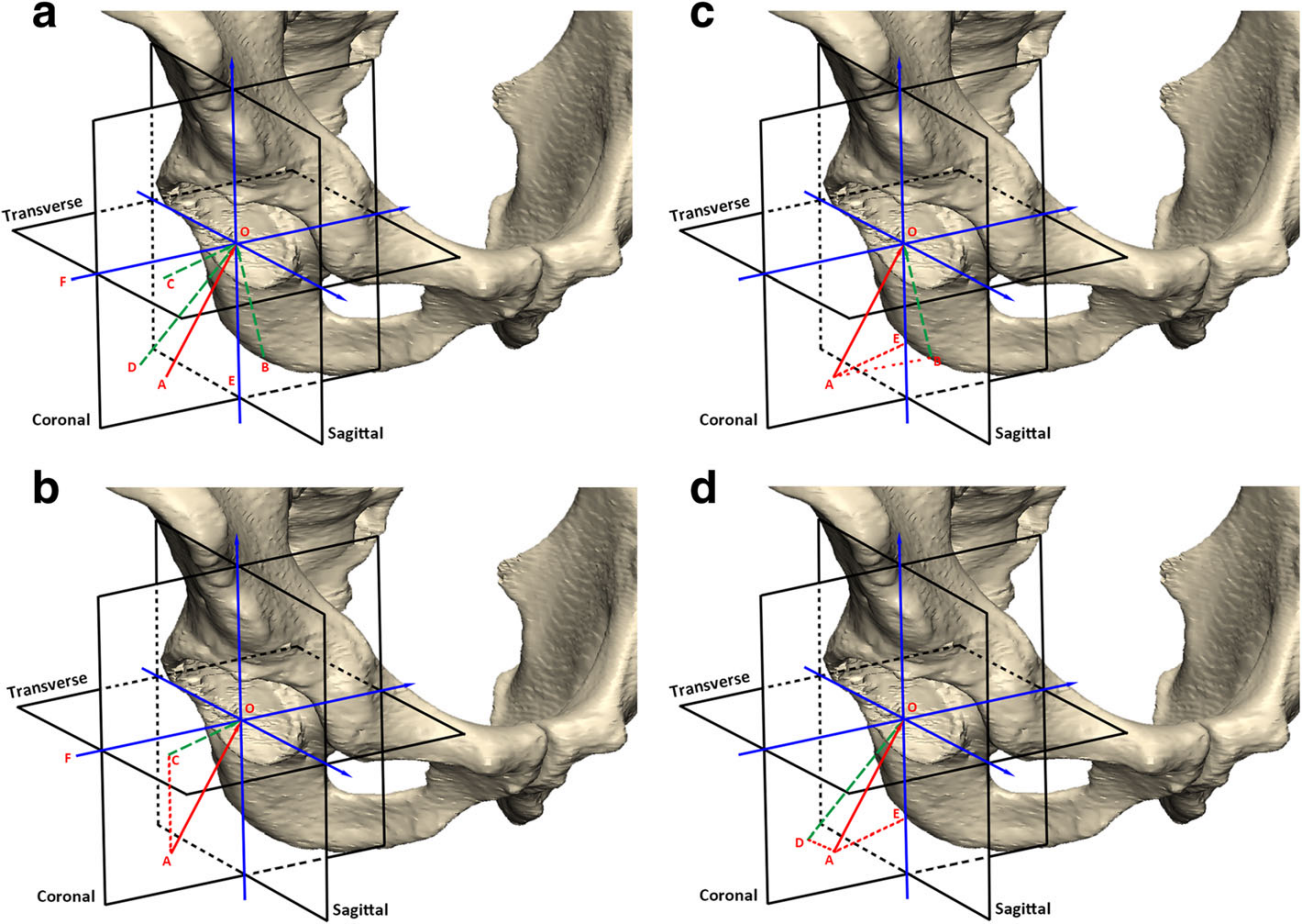
**a** Conception of Inclination and Anteversion in three different measurements of pelvic region.AO was pivot of the pelvic region; OE was longitudinal axis. The projection line of AO on sagittal plane was OB; OD was a projection line on coronal plane and OC was a projection line on transverse plane. **b** Anatomic Inclination (∠AOE) and Anatomic Anteversion (∠COF). **c** Operational Inclination (∠AOB) and Operational Anteversion (∠BOE). **d** Radiographic Inclination (∠DOE) and Radiographic Anteversion (∠AOD)

Murray in 1993 determined three methods for acetabular measurement, anatomical, operational and radiographic measurements of inclination and anteversion [9]. Anatomical inclination (AI) was the angle between the plane of acetabular face and the transverse plane, whose mathematical model was the angle between the acetabular axis of the patient and the longitudinal axis (Fig. 1b ∠AOC). Anatomical anteversion (AA) was the angle between the acetabular axis and the coronal plane when viewed in cranio-caudal direction. The mathematic model of AA was the angle between the acetabular axis that was projected to transverse plane and transverse axis (Fig. 1b ∠COF). Operative inclination (OI) was defined as the angle between the rod and operational bed in lateral position whose mathematical model was the angle between the acetabular axis and sagittal plane (Fig. 1c ∠AOB) [9]. Operative anteversion (OA) was known as the rotation of the rods around the transverse axis when inclination was determined. The mathematical model was the angle between the acetabular axis that was projected on to the sagittal plane and longitudinal axis (Fig. 1c ∠BOE) [9]. The radiographic inclination (RI) was defined as the face of the cup and transverse axis when projected on anterior-posterior view. It was the same angle between the acetabular axis projected on the coronal plane and longitudinal axis, alternatively (Fig. 1 ∠DOE). The radiographic anteversion (RA) was calculated by major and minor diameters of the ellipse which could be defined as the angle between the acetabular axis and the coronal plane (Fig. 1 ∠AOD) [10].

### 3D reconstruction and anatomical landmarks

**Fig. 2:**
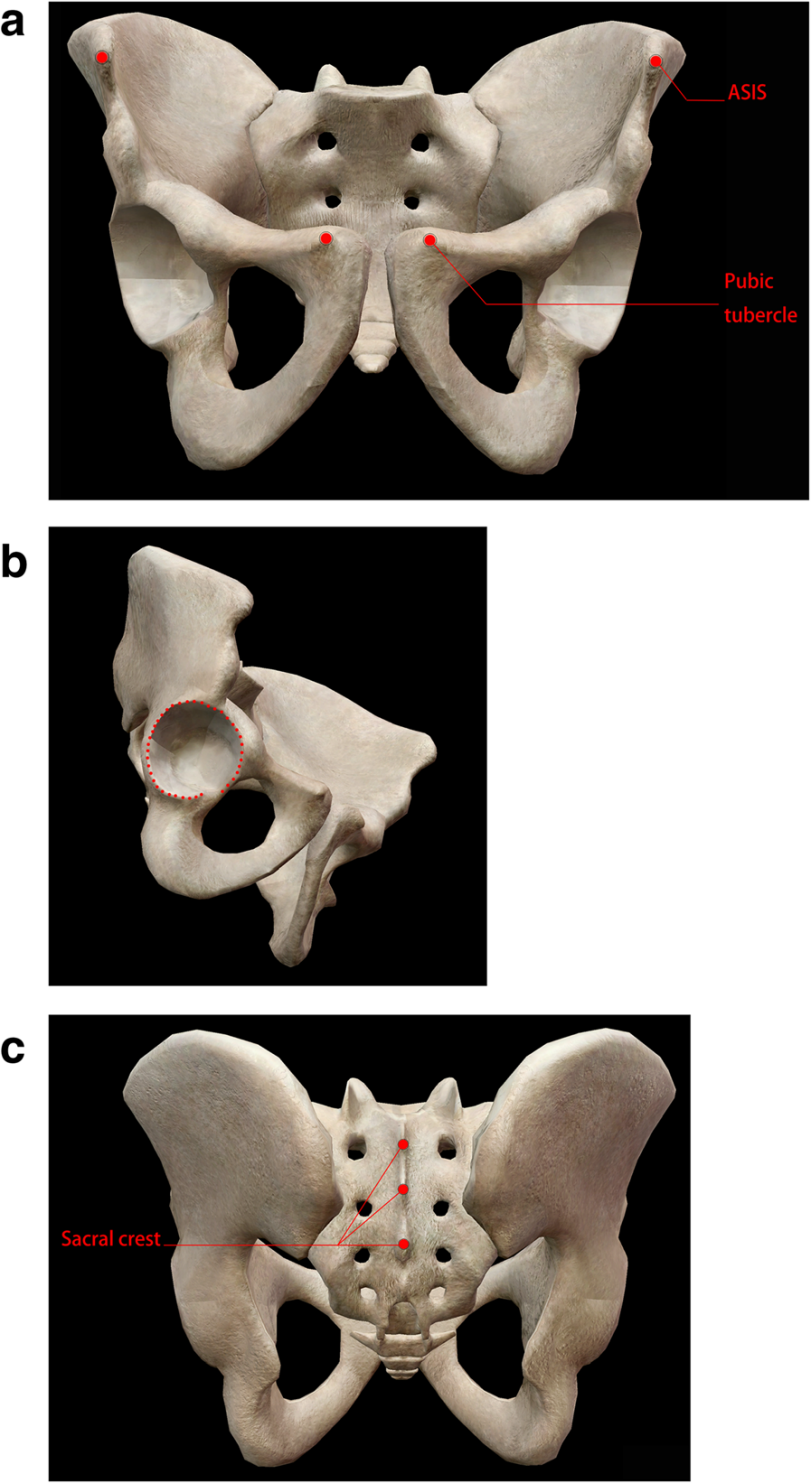
Bony landmarks, labelled for calculating acetabulum inclination and anteversion. **a** Label of ASIS and pubic tubercles. **b** Label of rim of acetabulum at least 30 points. **c** Label of sacral crests at least 3 points

After loading the DICOM data, 3D pelvic model was rebuilt by 3D–Slicer software (http://www.slicer.org/) and all landmarks on the pelvis were marked. (1) Acetabular rim: The rim of acetabulum except the gap of transverse acetabular ligament should be labeled, at least 30 points starting from the rim. Further, the distance between the adjacent points should be maintained in a coincident manner. (2) Bilateral anterior superior iliac spine (ASIS) and pubic tubercle. (3) Sacral crest: At least 3 points of sacral crest were labeled (Fig. 2). Landmarks were judged and labelled manually. One case was labelled by two orthopedists independently and the results were compared. If each landmark labelled by the orthopedists had a divergence less than 2 mm in case, the labelling work was considered as validation, otherwise another orthopedist was engaged for labelling. All bony landmarks were marked and the coordinates of each point (*x*, *y*, *z*) were exported for calculation.

### Determination of the acetabular axis

Hip joint was a hemisphere joint and normal vector of acetabular face (bottom) was named as the axis of acetabulum [16, 17]. The bottom of acetabulum was not flat. At least 30 points of acetabular rim were collected and these points were used to fit the acetabular bottom. Thus, the normal vector calculated was regarded as the axis of acetabula. The bias between the norm vector and acetabular axis was reported by Lubovsky, but did not show any significant result [18].

The method to calculate norm vectors was by minimizing the sum of the squares of the distances of all collection points. The Matlab software (MathWorks Inc., USA) used a formula called “fitNorm” which could be utilized to calculate the norm vector.

### Determination of reference planes

**Fig. 3:**
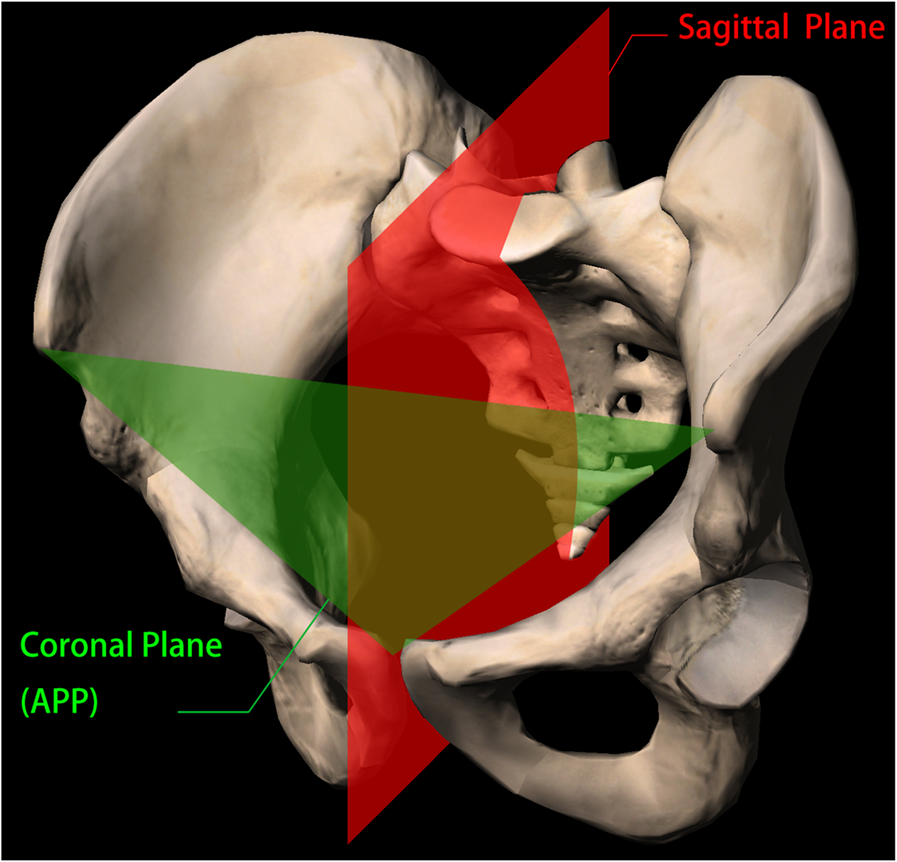
Coronal and sagittal plane that were defined as bony landmarks. Coronal plane (APP) was defined as bilateral ASIS and pubic tubercles. Sagittal plane was defined as midline of bilateral ASIS and sacral crest

Coronal plane was immeasurable in the pelvic CT model. However, when a body was placed in erect and supine position, anterior pelvic plane (APP) was almost parallel to the coronal plane [19]. As all cases were examined in supine position, we demarcated APP by bilateral ASIS and pubic tubercles (Fig. 3) [20]. Sagittal plane was much easier to determine as the plane was enclosed by midpoint of bilateral ASIS, and sacral crests were coincided with the sagittal plane (Fig. 3). Therefore, these coordinates were imported into the Matlab software. Norm vectors of coronal plane and sagittal plane were calculated by “fitNorm”.

Transverse plane was immeasurable in pelvic CT model either. As transverse plane was perpendicular to the coronal plane; mathematically, it meant normal vector of transverse plane was vertical to the normal vector of coronal. Using arrhythmic method, we subsequently calculated the formula of transverse plane:

$$ \mathrm{Norm}\left(\mathrm{Transverse}\right)=\mathrm{Vector}\left(\mathrm{ASIS}\right)\times \mathrm{Norm}\left(\mathrm{Sagittal}\right) $$

The perpendicular plane was calculated by three reference planes, which might due to APP that was almost paralyzed to the coronal plane. The angle between the two planes was calculated by [21]:

1 $$ \uptheta ={\mathrm{cos}}^{-1}\frac{\overrightarrow{n}\cdot \overrightarrow{m}}{\left|\overrightarrow{n}\right|\left|\overrightarrow{m}\right|} $$

$\overrightarrow{n}$ and $\overrightarrow{m}$ were two norm vectors of reference planes. If two planes were vertical, the angle calculated was 90°. The angle θ was examined by statistical methods with 90°.

### Calculation of inclinations and anteversions

We calculated these parameters strictly by definition and by manipulating the Matlab software. Normality test of each parameter was then examined to investigate whether these data could be used for further statistical tests, which were undetermined. Moreover, patients were classified by age and gender to confirm whether these factors affect distribution of acetabular degrees.

The angle was calculated by formula (1). For instance, OI was the angle between the acetabular axis and sagittal plane. Thus, $\overrightarrow{n}$ was vector of acetabular axis and $\overrightarrow{m}$ was vector of sagittal plane. For RI, which was the angle between the acetabular axis projected onthe coronal plane and longitudinal axis, the vector of acetabular axis should be projected to the coronal plane firstly. Vector of acetabular axis was regarded as two coordinates that started from P_1_(0, 0, 0) and ended with P_2_(*x*_2_, *y*_2_, *z*_2_). The formula of coronal plane was Ax + By + Cz + D = 0. Thus, the vector projected to the coronal plane was started from ${\mathrm{P}}_1^{\prime}\left(0-\mathrm{A}\bullet {\mathrm{t}}_1,0-\mathrm{B}\bullet {\mathrm{t}}_1,0-\mathrm{C}\bullet {\mathrm{t}}_1\right)$ and ended with ${\mathrm{P}}_2^{\prime}\left({x}_2-\mathrm{A}\bullet {\mathrm{t}}_2,{y}_2-\mathrm{B}\bullet {\mathrm{t}}_2,{z}_2-\mathrm{C}\bullet {\mathrm{t}}_2\right)$ [22].

2 $$ {\mathrm{t}}_1=\frac{D}{A^2+{B}^2+{C}^2} $$

3 $$ {\mathrm{t}}_2=\frac{Ax_2+{By}_2+{Cz}_2+D}{A^2+{B}^2+{C}^2} $$

### Statistical methods

Variable data were expressed as mean ± standard deviation and attributable data were expressed as percentage (%). Statistical significance was set at *p* < 0.05. Normality test of each measurement was analyzed by Kolmogorov-Smirnov test. Inclination and anteversion distributed by gender were examined by Student-t test and distributed by age group were examined by Chi-square test. To examine whether reference planes were vertical pair-wise, angles between two of the three reference planes were compared with 90 degrees and unpaired student-t test was applied. Analysis of Variance (ANOVA) was used to analyze the differences among inclinations and anteversions of anatomical operations and radiographic measurements, and pairwise comparisons were analyzed by least significant differences (LSD). All tests were analyzed via SPSS software, version 13.0 (IBM Inc., USA), which was provided by Huazhong University of Science and Technology.

## Results

### Basic information

A total of 100 cases were involved in the study and after excluding the ineligible cases a total of 61 cases continued further study. Five cases were excluded because of pool structure of ASIS caused by iliac crest graft. Seven cases were excluded after 3D reconstruction due to blurred anatomical structure by poor CT filming. Forty-nine cases (98 hips) were finally chosen.

The characteristics of gender and age distribution were presented in Table 1. In age distribution, we classified the patients based on age into sub-groups, less than 30 years old, 30–40 years old (40 excluded), 40–50 years old (50 excluded) and more than 50 years old.

**Table 1:** Characteristics of gender and age distribution

Gender Distribution		
Male	28 (of 49)	57.14%
Female	21	42.86%
Age Distribution		
Range of Age	35.69	
Mean of Age	18to 56	
Age Group		
< 30	18 (of 49)	36.73%
[30,40)	8	16.33%
[40,50)	18	38.78%
≥ 50	4	8.16%

### Vertical examination of reference planes

To examine whether these three reference planes were perpendicular, we analyzed the angles of three groups of reference planes, APP with sagittal, APP with transverse and sagittal with transverse. The angle between transverse and sagittal was 90° because transverse plane was transformed by sagittal plane. The angle between APP and sagittal was 88.4° ± 6.0° (*P* > 0.05). The angle between APP and APP and transverse was 83.2° ± 13.6° (*P* > 0.05). Therefore, all three reference planes were statistically perpendicular.

### Normality tests and comparisons among the three measurements

A normality test was performed to examine whether the three inclination and anteversion measurements were complied with the normal distribution. *P* > 0.05 was thought to comply with the normality test. All groups complied with normality distribution except RI with a significance of 0.001. After transformation by “Blom” Mode in SPSS, RI was complied with normality distribution.

After normality test, we then calculated the inclination and anteversion of different measurements with mean and standard deviations (SD). Transformed RI was involved in the calculation. Inclination and anteversion measurements were presented in Table 2.

**Table 2:** Inclinations and anteversions in different measurements and in gender and age distribution

	Total Samples	Gender Distribution			Age Distribution				
		Male	Female	Sig.	<30	[30,40)	[40,50)	≥50	Sig.
AI	37.48 ± 11.07	38.88 ± 12.15	35.62 ± 9.27	0.136	38.36 ± 11.57	41.01 ± 14.23	36.10 ± 9.73	33.03 ± 5.48	0.294
OI	45.12 ± 14.76	54.79 ± 16.34	46.89 ± 12.30	0.307	45.16 ± 16.14	45.16 ± 16.14	45.16 ± 16.14	45.16 ± 16.14	0.165
RI	48.76 ± 14.36	47.33 ± 29.81	52.39 ± 26.55	0.389	54.94 ± 29.13	34.28 ± 25.54	47.80 ± 28.38	63.5 ± 19.52	0.054
AA	18.12 ± 7.59	17.51 ± 7.98	18.93 ± 7.04	0.354	17.17 ± 7.25	19.99 ± 8.16	17.18 ± 7.89	23.06 ± 4.09	0.137
OA	24.97 ± 9.68	23.25 ± 9.53	27.25 ± 9.51	0.052	25.48 ± 9.44	25.48 ± 9.44	25.48 ± 9.44	25.48 ± 9.44	0.351
RA	14.30 ± 5.64	13.73 ± 5.92	15.06 ± 5.21	0.250	13.21 ± 4.73	13.21 ± 4.73	13.21 ± 4.73	13.21 ± 4.73	0.274

Inclination and anteversion were tested with gender classification. The results indicated that inclination and anteversion in both male and female showed no significant differences (*P* > 0.05). This illustrated that gender classification did not influence the distribution of inclination and anteversion. Test of divergence with age span was also calculated, which showed that the age span mentioned above did not interfere with the distribution either. All these results proved that the parameters of acetabula were kept stable in population. Inclinations and anteversions in gender and age distribution were presented in Table 2.

**Fig. 4:**
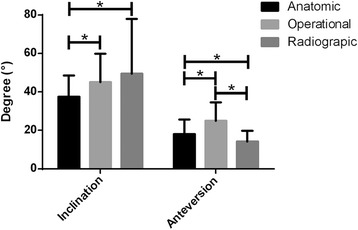
Measurements of inclination and anteversion of anatomic, operational and radiographic measurement, * *P* < 0.05

Subsequently, the difference among the inclinations in anatomic, operative and radiographic measurements was confirmed. Results of ANOVA illustrated significant differences within AI, OI and RI (*P* < 0.01). Comparison of LSD found that AI showed significant differences with OI and RI (*P* < 0.05), while no significant differences between OI and RI (*P* > 0.05) were observed (Fig. 4). We also tested the divergence in anteversions by three measurements, which showed significant difference among AA, OA and RA (*P* < 0.01) and with a significant divergence in LSD (Fig. 4).

## Discussion

Our research used the 3D reconstruction and mathematics method, which presented the deviations in the inclinations and anteversions in the three measurements. Inclination results showed that value of RI was greater than OI, (*P* < 0.05) and was progressively greater than AI (*P* < 0.05), (Fig. 4). No significant difference was observed between OI and RI (*P* > 0.05). Anterversion measurements presented results where OA was greater than AA (*P* < 0.01) and was more progressive than RA (*P* < 0.01).

Bone landmarks were used to determine reference planes and these in turn help the measurements to be more precise. Several research studies have demonstrated that transverse plane of CT was used to measure the inclinations and anteversions [23]. The reference plane was the scale of CT device. The measurement was less precise when using CT as a reference, because the position bias of the patient. A small divergence in the position hinders the coincidence of transverse plane of the patient and device, as well as coronal and sagittal planes. As a consequence, bias occurs when using device reference while reference planes in our research minimized the position bias.

The results of our research illustrated that inclinations and anteversions in the operation were different from those on the films. Anteversion at AP site was larger than the anteversion that was determined by lateral approach (OA > RA). The divergence of OA and RA was 8.946° ± 6.618°. As the acknowledged anteversion on AP site was from 15 to 20 degrees, the ideal anteversion examined during operation should raise for about 9 degrees. Inclination on anterior approach was less than it was in AP site (RI > AI) and anteversion on anterior approach was larger than it was in AP site (AA > RA). In the same way, the difference of RI and AI was 8.020° ± 7.313° and difference of AA and RA was 3.738° ± 2.235°, meaning that inclination in anterior approach should be adjusted about 8 degrees lesser while anteversion adjusted 4 degrees larger. These divergences might lead to operator’s confusion.

Parameters that were measured from 3D reconstruction model seemed to be much closer to the anatomical measurements because the 3D pelvic model restored the anatomical structure of the pelvis. Humbert et al. investigated the inclinations based on 3D pelvic model and reported a result of 36° (30–40°), and these results were confirmed by few other investigators. Additionally, our research reported that the operational inclination was 45.12° with a deviation of 14.76° and anteversion was 24.97° ± 9.68°. An investigation among east Chinese population confirmed with our findings, showing the divergence among three measurement of acetabular orientation [24].

Although anatomical, operational and radiography measurements belonged to different means of measurements, Murray provided conversional formulae for different methods [9, 25]. For instance, tan*OA* = sin *RI* ∙ cos *RA*, tan*AA* = cos *OI* ∙ cos *OA* and tan*AA* = cos *OI* ∙ cos *OA*. We examined our results with these formulae. The result of *OA* − tan^−1^(sin*RI* ∙ cos *RA*) was 11.20° ± 8.12° (*P* < 0.05), *AA* − tan^−1^(cos*OI* ∙ cos *OA*)was −12.77° ± 8.44° (*P* < 0.05) and *RA* − sin^−1^(tan*OI* ∙ cos *OA*) was −26.63° ± 19.48° (*P* < 0.05). These calculations were in contrast with the alterations provided by Murray. Therefore, it was impossible to transform inclinations and anteversions of one measurement to another.

Although divergence of inclinations and anteversions in anatomical, operational and radiographic measurements prevailed in our study, there were still few drawbacks in our study. It was out of anticipation that RI did not comply with the normality test, although it was transformed to normality. Also, inclinations and anteversions were influenced by the physiological curvature of lumbar spine [26]. When the lumbar physiological curvature was straight, anteversion and abduction turned to be lesser; nevertheless, as the curvature increased, anteversion and abduction turned to be larger [6, 27]. With the growing age, lumbar spine appears to be degenerated to some degree and a change in the physiological curvature was observed. Therefore, abduction and anteversion were certainly under influence. Researches had already reported that abduction and anteversion were changed regularly with the influence of age, and this change might be due to the factors associated with lumbar spine [12].

## Conclusion

Our research revealed that there were deviations among anatomical, operational and radiographic measurements of acetabulum. OA was about 9 degrees larger than RA. RI was about 8 degrees larger than AI and AA was about 4 degrees larger than RA. These divergences might in turn interfere with the operator’s decision.

## Abbreviations

CT: Computer tomography
DDH: Developmental dysplasia of the hip
FAI: Femoroacetabular impingement syndrome
LSD: Least significant differences
OA: Operative anteversion
SD: Standard deviations
THA: Total hip arthroplasty
